# Book Reviews

**Published:** 1861-04

**Authors:** 


### OUR LIBRARY TABLE.

*The Forms, Complications, Causes, Prevention, and Treatment of Consumption and Bronchitis, comprising also the Causes and Prevention of Scrofula.* By James Copland, M.D., F.R.S., F.R.C.S., &c. (Longman).—In this work the valuable treatises on Pulmonary and Laryngeal Consumption, which originally appeared in the author's *Dictionary of Practical Medicine*, are republished with considerable additions. The treatise on Bronchitis is to all intents and purposes a new one. Dr. Copland's writings and their great value are too well known to the profession to need any critical comment from the reviewer. If a special indication of the peculiar importance of the foregoing work be necessary, it is best derived from the following prefatory observations of the author:—

"In the treatises on the above important diseases I have endeavoured to assign with precision the several forms and states they present, and with due reference to the conditions of vital force (of vital energy, power, or endowment). This force, in its various and ever-varying manifestations, throughout the general systems and special organs, animal and organic, of the body, has been made, in connexion with the states of vascular action, the basis of therapeutical indications for the diseases comprised in this work, conformably with the principles of pathology and medical practice adopted and developed in my *Dictionary of Practical Medicine.* While discussing the pathology and treatment of Consumption and Bronchitis in the present publication, not only the conditions of vascular action and of the circulating fluids, but also the manifestations of vital force or power by which these conditions are influenced, developed, and controlled, are considered as fully as the difficulty of these subjects permit; for in actual practice the successful treatment of these diseases chiefly depends upon a correct estimate of these most important pathological conditions. During the last half century physicians have had their minds pre-occupied, and their attention carried away, by the recognition of changes of structure and of palpable organic alterations, from a due estimation of those conditions of vital force, of vascular action, and of the circulating fluids, which constitute the essential principles of disease, which chiefly engaged the deepest consideration of their predecessors, and which guided them to the adoption of appropriate indications and means of cure, and to practical results at least as successful as those achieved by the modern pathologist."

*Infant Feeding and its Influence on Life, or the Causes and Prevention of Infant Mortality.* By C. H. F. Routh, M.D., M.R.C.S.L., M.R.C.S., Physician to the Samaritan Free Hospital for Women and Children. 1860 (Churchill).—This work, we have little doubt, will prove very acceptable both to the profession and the public. The question of Infant Mortality has of late, thanks to the influence exercised by the Social Science Association, been brought very prominently into notice. It is now pretty generally admitted that the high rate of death, too commonly found to prevail among infants, is a subject involving questions not merely of medical but of great public interest. Dr. Routh, in this work, has examined these questions in their most important bearings, and with particular reference to the influence of *improper feeding*, as a cause of mortality among children of tender age. He shows by interesting statistical data the baneful effects of this cause upon infant life; and he discusses at large the principles which should govern, and the right method of carrying out, infant feeding. Those chapters of the work which refer to the employment of wet-nurses, and the indirect influence of wet-nursing upon infant mortality, will be read with great interest. For the rest, the practical value of the work is of very high grade, and we heartily commend it to the notice of the profession. The following extract will show the interesting fashion in which Dr. Routh deals with his subject:—

"The whole analogy of comparative anatomy proves that all young animals require animal food for some time after birth, because this, or some adventitious animal structure, is generally supplied by the parent. The infant itself is so anatomically and physiologically made as to be capable of digesting animal food only.

"In many species of mollusca, and especially in gasteropoda, in many insects, and among the batrachian reptiles, the mother produces, together with the egg, what is called a nidamentum, which nourishes it for some time after its birth. Certain insects even feed upon the external envelopes which surround them, as in the case of the *stratismys chameleon.*

"The yellow substance which surrounds the abdominal parietes in some animals, or which is enclosed in the central abdominal cavity, is an auxiliary of this kind. Its presence explains the fact that spiders and snakes, for instance, remain some time after birth without requiring any other kind of food. The raw food which the greater number of birds give to their young is exclusively animal; hence the more readily obtainable and digestible. The northern ducks and the petrels, with their nests situated on high rocks near the sea, easily procure this food, and they always return to their nests richly laden with fish. The sparrows nourish their young with insects and worms, which they find everywhere in abundance; and hence certain rapacious birds, which require a greater amount of animal food for their young, become at the breeding season particularly audacious in order to procure it.

"Some of the sparrow and crow tribe bring the nourishment in their beaks, emptying it into those of their young. The rapacious birds, on the contrary, bring it in their claws, place it before their young, and tear it in small pieces for them. The heron and the pelican bring the fish in the pharynx, which is dilated to a large pouch below the bill; and the pelican applying its lower jaw against its own breast, allows its young to eat out of this pocket as out of a plate. Among some species of vultures and dark-winged eagles, the crop seems to serve as a reservoir for the food intended for the young. Approximating to a higher degree of maternal co-operation, the female does not give nourishment to her young till she has in part digested and assimilated it. The bees and wasps are of this class, and swallow some pollen, and then disgorge it mixed with honey. Among pigeons, the greater number of *grallatores*, some *palmipedes*, and many sparrows, the mucous membrane of the œsophagus is dilated into a crop, well supplied with vessels, into which the grain which is difficult to digest is first conveyed, and then softened under the chemical influence of a fluid analogous to the gastric juice of the stomach. When half-digested, and converted into a kind of chyme, it is subsequently disgorged into the beak of their young. This modified chyme it is which is popularly called pigeon's *milk.* The male assists in this operation as well as the female. Finally, in mammalia we arrive at the production exclusively by the mother, of milk, which bears in its composition considerable resemblance to the diluted yolk of egg, and in some respects to the nidamentum. It will be seen from the preceding review that the food which is required by the young is essentially animal; and in those cases even where the birds themselves are granivorous, or vegetable feeders, they either supply their young with animal food exclusively, or else with vegetable food so semi-digested in, or so intermixed with, the animal fluids, that for all purposes it may be regarded as animal food.

"Gradually as the young animal becomes older, this exclusive dependence upon the maternal supply ceases. Among pigeons, for instance, after three days the young bird begins to partake of other food also. The reindeer, at the end of some days, begins to eat grass and lichens; and the calf in about three weeks can no longer live exclusively on its mother's milk, but requires other food. Still the dependence of young animals upon the food which they directly obtain from the mother in the natural state, is very close. In the case of the *simia rhesus*, that animal attaches itself to its mother's nipple, and remains in this position for fifteen days, in sleeping as well as waking, never leaving one breast but to attach itself to the other. To endeavour, therefore, to nourish any young animal exclusively on vegetable food, is contrary to the entire law of nature, and especially so in man, where the parental relations are so much closer, and maintained for so much longer a period." (pp. 132-5.)

*On the Origin of Species by means of Organic Affinity.* By H. Freke, A.B., M.B., M.D., T.C.D, M.R.I.A., &c. (Longman.)—We dare not suffer ourselves to be seduced into those broad paths of philosophical speculation in which Dr. Freke journeys. The following extract from the preface to his work may, however, be quoted as an index of its character:—

"I cannot refrain from expressing the great satisfaction I have felt on recognising a coincidence between one of the ablest living naturalists and myself upon *one* important question—and I regret that it should be *only* upon one—in relation to this interesting inquiry. I refer to the fact that both Mr. Darwin and myself have been led—each by his own peculiar views—to believe that all organic creation has originated from a single primordial germ.

"In directing attention to this coincidence, I desire that it should be most distinctly understood that nothing could be more remote from my intention, than to attempt in the slightest degree to detract from the originality of that distinguished author's able work. We had both reached the same result through a totally different channel. Mr. Darwin attained by *analogy* to what I had attempted to establish by *induction;* and it is of importance to science that naturalists should be aware that such is the case. For the fact of two independent inquirers, *utterly unconscious of each other's existence*, having reached, by *a totally different order of inquiry*, an *identical* and at the same time an *unlooked for result*—at least on my part altogether unlooked for—such fact, I say, impresses that conclusion with such a stamp of probability as *almost*, in my mind, to withdraw it from the domain of *hypothesis.*

"I shall here merely add, that nothing is advanced in this publication that is not perfectly in harmony with the Mosaic record of creation."

*The Philosophy of Insanity.* By a late Inmate of the Glasgow Royal Asylum for Lunatics at Gartnavel (Edinburgh: Maclachlan and Stewart, 1860.)—This highly interesting work is written by the author as a grateful acknowledgment of the good which he himself had received, from time to time, in a lunatic asylum; and in the hope that the record of his experience would tend to diminish the popular fear entertained of those institutions. "I have endeavoured," he says, "to strip lunatic asylums of all imaginary terrors, and to render them familiar to the mental view; and, by so doing, I hope that I may be instrumental, in some cases, in doing away with the necessity for their use. This has been a natural consequence in corresponding cases, and I know no reason why lunacy and lunatic asylums should form exceptions to the general law." (p. 14.) The singular vigour and interest of the work in many parts, but particularly where the author is recounting some portions of his own history, may be judged of by the following quotations:—

"Tobacco, if long made use of, takes a fearful hold on the mind and body. The sudden deprivation of it is a desperate punishment, and has in many cases produced temporary madness. The magistrate who condemns two offenders for the same offence to the same term of imprisonment—the one a slave to tobacco, the other free from its dominion—condemns them to a very unequal amount of punishment. There are two circumstances to be considered in connexion with this case. One may be termed physical, and the other mental; there is the morbid craving for the accustomed supply of the drug, and there is the habit formed by the furnishing of that supply. Every smoker must have observed that during sickness, when the desire for tobacco, and very likely for everything else, had left him, that the mere force of habit would keep impelling him to fill and light his pipe. It would require hard fighting to conquer either of these habits, but when united they will be found in most cases invincible.

"The late Mr. Leith, coach proprietor in Glasgow, had a groom who could not be contented without a brass pin in his mouth, with which he indulged in the remarkably cheap luxury of jagging his gums. One day Mr. Leith had occasion to send him to Hamilton, distant about ten miles, when he said to his man, 'Now, Tom, if you will ride to Hamilton and back without the pin in your mouth, I will give you five shillings.' Tom threw the pin from him, mounted his horse, and rode off with a face that had quite a conquer-or-die look about it. To keep himself free from temptation, he also threw away a few spare ones that he had in the breast of his jacket. He returned crestfallen, and confessed that he had been obliged to dismount on this side of Hamilton, and regale his gums with a thorn from a hedge, as a substitute for the accustomed pin. Habit had conquered; indeed, I believe that habit almost always gains the first, second, and perhaps the third battle; but fight on, and perseverance will annihilate him.

"When a young man, I was at one time employed close upon the sea shore, and having little companionship, I attached myself very closely to my pipe; and the consequence was that I smoked myself into a low, nervous, feverish state, besides getting nearly blind. I felt that I ought to refrain, but the desire and the habit swept the judgment and the will before them as the autumnal wind sweeps before it the rustling, dry, brown forest leaves.

"At a short distance from where I dwelt there was a rock, the base of which was dry at low water, but a depth of six or seven feet was around it at flood. In a cleft of this rock I deposited my pipe at low water, resolving that I would drop smoking if I could, and that, at any rate, I would not take a smoke till I came back and took the pipe from its hiding-place in the rock. This, of course, I meant as a check upon me, and so it was for a few hours. I faced it boldly for about four hours. The consciousness of doing right upheld me, and then habit began to turn my pockets inside out, in search of something which I vainly strove to make myself believe was not the pipe. Then came the physical craving, the unbearable gnawing, and the two kept dragging me back to the rock, as the dog drags from his box the very unwilling badger. I spent a miserable afternoon and night, got silent and sulky, and very sententious in my mode of expressing myself. For example, my landlady kindly asks, 'Are you no very weel the nicht, my man?' 'Quite,' says I. 'I havena seen you smoking this while,—hae you nae tobacco?' 'Plenty,' replies I. 'Dear me, but you are as short as cat's harns,' says the good old woman, and so ends our conversation.

"I went to bed, but could not sleep. About midnight strange ideas began to flit through my brain. I could stand out no longer. On goes my clothes, with very little ceremony as far as regarded braces and buttons, and off goes I post haste for the rock. The tide was about half run, but a strong and steady breeze from seaward was still dashing the waves far up upon the rock, sparkling with that phosphorescent gleam peculiar to salt water when stirred by whatever cause in darkness. In went I,—the second and third waves which met me, dashed up breast high and filled my mouth with brine; but wave number four found me under the lee of the rock with my pipe in my hand, and before many minutes had elapsed I was smoking away furiously, with my boots full of very cold water, and my clothes hanging about me like wet sails." (p. 32.)

"Lunacy, like rain, falls upon the evil and the good; and although it must for ever remain a fearful misfortune, yet there may be no more sin or shame in it than there is in an ague fit or a fever.

"With a feeling allied to fear we behold a grim array of the insane dead—once famed in science, in arts, in literature, in arms—as it were starting from their graves and passing in review before us. How our hearts cling to Cowper, with his pale, pensive face, and mild, warm heart, that throbbed and glowed with love to all that nature ever bore. And how we shrink as crimson-coloured Clive, with martial step and eye of pride, strides past lacquered with Eastern blood. And slowly rising from her sun-scorched grave, glides past the much-loved L. E. L., spiritual as in the days when she made young hearts to thrill under the witching spell of her melody—her whose genius, in our youthful days, we worshipped unseen:—The heart-stilling liquid is in her hand—her eyes are turned upwards—she prays for forgiveness, and fancies that she hears the far, far distant notes of an angel voice mingling with the deep breathings of her fearful despair. And, 'revisiting the glimpses of the moon,' conscientious and stern, stands Miller, who died nailing the white flag of science to the crimson shoulder of the cross. And thou Tannahill, sweet songster of the west, with thy sensitive nature, which shrunk from the briars and nettles which pricked and stung thy tender feet; what a sympathetic chill creeps round our heart as we look upon thy wet, shivering form, and hear the night wind stirring the drenched bay leaves which encircle thy pale and dripping brow. No man of fire or blood wert thou; and, true to thy nature, thou chose the love-mad maiden's death, who drowns her hopeless grief, closes her sleepless eye, and cools her burning brain beneath the stream. How sad, how sorrowful to think that a mind which has shed light and joy into many a heart and home, should itself disappear amid despair and darkness. And, glancing like a meteor in the gloom, shines Goldsmid's jewelled form: insanity's blast blows hard—his golden anchors are dragging—a crimson winding-sheet flaps in the gale, and an open sepulchre lies under his lee.

"To this dread appeal also answers Irving, Swift, Collins, Castlereagh, Chatterton, Hall, Romilly, Defoe—'What! will the line stretch out to the crack of doom?'" (p. 91.)

*Hints on Insanity.* By John Millar, L.R.C.P. Edin., Medical Superintendent, Bethnall House Asylum, London. (Renshaw.)—These hints are intended to be "useful to those medical men who have had no opportunity, during their professional education, of becoming practically acquainted with Insanity, and whose time is too much occupied to permit them to make a special study of a disease which they are seldom called upon to treat." The work is well fitted to effect its object, and will be found very serviceable to medical men who are only rarely called upon to certify in cases of lunacy.

*On Insufficiency of the Aortic Valves in connexion with Sudden Death; with Notes, Historical and Critical.* By JOHN COCKLE, M.D., F.L.S., Physician to the Royal Free Hospital, pp. 30. (Davies.) —This is an exceedingly interesting and thoughtful essay, the nature of which is fully expressed in the title.

*De la Colonie de Fitz-James succursale de l'Asile Privé d'Aliénés de Clermont (Oise) considerée au point de vue de son organisation Administrative et Medicale.* Par le Dr. GUSTAVE LABITTE, Medicin en Chef de l'Etablissement. Paris, 1861. (*Baillière.*)—Dr. Labitte gives an interesting account, and reports most favourably, after a four years' trial, of the success of this colony. From this experience he deduces the following general principles of asylum construction and management:—

"A Lunatic Asylum ought to suffice in itself, that is to say, it ought to have in its patients, by a wise application of such services as they can give, all the means of diminishing expenditure. For this purpose a very large asylum population is necessary, because in a great number of patients it is easy to find workers suited to all the wants of the establishment. The importance of this population permits also the formation of a farm, an indispensable creation not only for the treatment of the patients but also for profitable management. The farm ought to be organized upon a sufficiently large scale, because all cultivation of this kind is less expensive the more extended it becomes. A Lunatic Asylum ought then to enclose at least a thousand patients of both sexes. From this population it will be easy to select, apart from the patients employed in the workshops of the establishment, two hundred lunatics to work upon the farm. This number is sufficient for the cultivation of two hundred hectares of arable land, an amount indispensable to the wants and alimentation of such an asylum. This population, moreover, permits more frequent changes between the asylum and the farm—changes always favourable to the patients and often necessary for the order and discipline of this last establishment. The asylum and the colony ought to be dependent upon one administration alone, of which the centre should be at the asylum. Lastly, in order that the services should suffer no obstacle in their execution, there should be but one head, and that head should be the physician." (p. 23.)

The interesting and useful *Account of Sir Charles Bell's Discoveries in the Nervous System*, by Mr. ALEXANDER SHAW, Surgeon to the Middlesex Hospital (Murray), and appended to the sixth edition of the Bridgewater Treatise on the "Hand," has been published by the author in a separate form. We have also to notice the publication of a third edition of Mr. Charles Bray's suggestive work on the *Education of the Feelings or Affections* (Longman); and a second edition of Dr. Guy's *Principles of Forensic Medicine* (Renshaw). This excellent work we shall recur to in a subsequent number, but in the meantime we may state that, although some portions of it have been abbreviated and others omitted, yet several new subjects have been added, also many woodcuts—a novel feature in English works on Medical Jurisprudence. Written originally for students, and this object being kept in view in the present edition, the utility and value of the work as a class-book are greatly enhanced by its altered form.

